# County-level barriers in the COVID-19 vaccine coverage index and their associations with willingness to receive the COVID-19 vaccine across racial/ethnic groups in the U.S.

**DOI:** 10.3389/fpubh.2023.1192748

**Published:** 2023-10-12

**Authors:** Jessica R. Fernandez, Paula D. Strassle, Jennifer Richmond, Vickie M. Mays, Allana T. Forde

**Affiliations:** ^1^Division of Intramural Research, National Institute on Minority Health and Health Disparities, National Institutes of Health, Bethesda, MD, United States; ^2^Division of Genetic Medicine, Department of Medicine, Vanderbilt University Medical Center, Nashville, TN, United States; ^3^Departments of Psychology and Health Policy and Management, UCLA Fielding School of Public Health and the UCLA BRITE Center for Science, Research and Policy, University of California, Los Angeles, Los Angeles, CA, United States

**Keywords:** COVID-19 vaccination intentions, health disparities, race/ethnicity, structural barriers, county-level vaccination barriers, COVID-19 preventive health services

## Abstract

**Background:**

County-level vaccination barriers (sociodemographic barriers, limited healthcare system resources, healthcare accessibility barriers, irregular healthcare seeking behaviors, history of low vaccination) may partially explain COVID-19 vaccination intentions among U.S. adults. This study examined whether county-level vaccination barriers varied across racial/ethnic groups in the U.S. and were associated with willingness to receive the COVID-19 vaccine. In addition, this study assessed whether these associations differed across racial/ethnic groups.

**Methods:**

This study used data from the REACH-US study, a large online survey of U.S. adults (*N* = 5,475) completed from January 2021-March 2021. County-level vaccination barriers were measured using the COVID-19 Vaccine Coverage Index. Ordinal logistic regression estimated associations between race/ethnicity and county-level vaccination barriers and between county-level vaccination barriers and willingness to receive the COVID-19 vaccine. Models adjusted for covariates (age, gender, income, education, political ideology, health insurance, high-risk chronic health condition). Multigroup analysis estimated whether associations between barriers and willingness to receive the COVID-19 vaccine differed across racial/ethnic groups.

**Results:**

American Indian/Alaska Native, Black/African American, Hispanic/Latino ELP [English Language Preference (ELP); Spanish Language Preference (SLP)], and Multiracial adults were more likely than White adults to live in counties with higher overall county-level vaccination barriers [Adjusted Odd Ratios (AORs):1.63–3.81]. Higher county-level vaccination barriers were generally associated with less willingness to receive the COVID-19 vaccine, yet associations were attenuated after adjusting for covariates. Trends differed across barriers and racial/ethnic groups. Higher sociodemographic barriers were associated with less willingness to receive the COVID-19 vaccine (AOR:0.78, 95% CI:0.64–0.94), whereas higher irregular care-seeking behavior was associated with greater willingness to receive the vaccine (AOR:1.20, 95% CI:1.04–1.39). Greater history of low vaccination was associated with less willingness to receive the COVID-19 vaccine among Black/African American adults (AOR:0.55, 95% CI:0.37–0.84), but greater willingness to receive the vaccine among American Indian/Alaska Native and Hispanic/Latino ELP adults (AOR:1.90, 95% CI:1.10–3.28; AOR:1.85, 95% CI:1.14–3.01).

**Discussion:**

Future public health emergency vaccination programs should include planning and coverage efforts that account for structural barriers to preventive healthcare and their intersection with sociodemographic factors. Addressing structural barriers to COVID-19 treatment and preventive services is essential for reducing morbidity and mortality in future infectious disease outbreaks.

## Introduction

In response to the SARS-CoV-2 (i.e., COVID-19) viral outbreak, the United States (U.S.) released three effective COVID-19 vaccines in December 2020 and March 2021 (1). Despite the protection/reduction from severe morbidity offered by the COVID-19 vaccines, a significant portion of the U.S. remained unvaccinated by the end of 2021 (2). Moreover, initial COVID-19 vaccination rates varied across racial/ethnic groups. Between December 2020 and April 2021, American Indian/Alaska Native, Black/African American, Hispanic/Latino, Multiracial, and Native Hawaiian/Pacific Islander adults had lower vaccination rates compared to Asian and White adults (3).

Disparities in COVID-19 vaccination uptake were not unexpected. Prior to the release of the COVID-19 vaccines, experts warned of racial/ethnic disparities in COVID-19 vaccination due in large part to the long history of racial/ethnic inequities within the U.S. healthcare system (4). In the early phase of the COVID-19 vaccination rollout, for example, healthcare facilities in metropolitan counties with higher proportions of Black residents and rural counties with higher proportions of Hispanic residents were less likely to serve as COVID-19 vaccine administration locations (5). Moreover, many individuals from marginalized racial/ethnic groups were hesitant to take the COVID-19 vaccine given their historical mistreatment by the healthcare system (e.g., historic exploitation of Black/African American and American Indian/Alaska Native populations in biomedical studies). Nationally representative surveys of U.S. adults conducted prior to the release of COVID-19 vaccines reflected these concerns. Black/African American and American Indian/Alaska Native adults, and in some cases, Hispanic/Latino adults, reported lower intentions to vaccinate compared to White adults (6–8). In many cases, individual-level factors were associated with COVID-19 vaccination intentions including beliefs about the vaccine, such as concerns over side effects and/or the rushed development of the COVID-19 vaccine (9, 10).

In addition to individual-level factors, public health-oriented studies examined the association between structural factors, such as county-level characteristics, and COVID-19 vaccination intentions (11–14). For example, the Centers for Disease Control and Prevention (CDC) COVID-19 Response Team examined the association between COVID-19 vaccination and county-level vulnerabilities measured by the CDC Social Vulnerability Index (i.e., “SVI,” a composite measure including socioeconomic status, household composition and disability, racial/ethnic minority status and language, and housing type and transportation) (13). In the first 3 months of the U.S. vaccination program (i.e., December 2020–March 2021), counties with higher scores on the SVI had lower COVID-19 vaccination rates (13). Studies also found that counties' SVI scores were associated with individuals' COVID-19 vaccination intentions (11).

These studies using the SVI highlighted the impact of social vulnerabilities on COVID-19 vaccination intentions. Additionally, public health scholars emphasized the importance of both social vulnerabilities as well as healthcare system barriers when assessing structural factors associated with COVID-19 vaccination (15). In February 2021, the CDC released a second composite measure developed by Surgo Ventures that extended the SVI (16). The COVID-19 Vaccine Coverage Index (CVAC) included U.S. county-level scores of social vulnerabilities (i.e., sociodemographic barriers captured by socioeconomic disadvantage and lack of access to information) and the healthcare system (i.e., limited healthcare system resources, healthcare accessibility barriers, and irregular care-seeking behaviors). The CVAC also included each county's history of receiving various vaccines (i.e., history of low vaccination captured by histories of lower coverage and high refusal rates).

Given that the CVAC is a newly developed tool, few studies have used it in the literature (17–20) and studies have yet to look at the impact of the individual CVAC components on vaccine willingness. The present study linked county-level barrier scores on the CVAC with survey responses from a large, diverse panel of U.S. adults who reported their willingness to receive the COVID-19 vaccine. The study aims included examining whether (i) county-level vaccination barriers varied across racial/ethnic groups, (ii) county-level vaccination barriers were associated with willingness to receive the COVID-19 vaccine and (iii) the association between county-level vaccination barriers and willingness to receive the COVID-19 vaccine varied across racial/ethnic groups.

## Materials and methods

### Data source

The present analysis used data from the REACH-US (Race-Related Experiences Associated with COVID-19 and Health in the United States) study. The REACH-US study is a cross-sectional online survey of adults living in the U.S. recruited from an existing opt-in survey panel hosted by YouGov, a non-partisan research firm. Participants completed the survey between January 26, 2021 and March 3, 2021.

Participants were initially recruited to YouGov's proprietary survey panel using online advertising, email communication, and partner-sponsored solicitations (~1.8 million U.S. panel members). Eligible YouGov panel members were proximity matched to the 2018 American Community Survey 1-year sample based on race/ethnicity, gender, age, education level, and language preference (English Language Preference or Spanish Language Preference for the Hispanic/Latino subgroup only). The target sample consisted of 500 American Indian/Alaska Native, 1,000 Asian, 1,000 Black/African American, 1,000 Hispanic/Latino, 500 Multiracial, 500 Native Hawaiian/Pacific Islander, and 1,000 White adults (total *N* = 5,500).

This quota sampling method was implemented to increase diversity and facilitate comparisons across racial/ethnic groups. Eligible participants for the REACH-US study were invited by email by YouGov, completed their survey responses online, and received panel rewards and/or incentives for their participation. Once quotas were met for each racial/ethnic group, sampling weights were calculated for REACH-US study participants. Race/ethnicity-specific multivariable logistic regression models, adjusting for age, gender, years of education, and region were used to estimate probability of inclusion in the study. Propensity scores were then grouped into deciles and post-stratified on age, gender, years of education, region, language preference (Hispanic/Latino only), and 2020 and 2016 Presidential vote choice (used to correct for sampling bias based on political affiliation). This matching and weighting approach generated a final sample weight for each participant and allowed for generating nationally representative estimates within each racial/ethnic group.

A complete case analysis was used in which participants were excluded if they had missing data on any study variables [county-level vaccination barrier (CVAC) score (*n* = 1), age (*n* = 2), annual household income (*n* = 13), health insurance coverage (*n* = 8), and political ideology (*n* = 1)]. The final sample included in the analysis was 5,475 participants. Given that YouGov provided deidentified data to the study team, this study was considered exempt, non-human subjects research as determined by the Institutional Review Board at the National Institutes of Health.

### Measures

#### County-level vaccination barriers

County-level vaccination barriers were drawn from the CVAC (developed by Surgo Ventures and made available by the CDC, https://data.cdc.gov/stories/s/Vaccine-Hesitancy-for-COVID-19/cnd2-a6zw/) and linked with data from the REACH-US study using the U.S. ZIP Code reported by REACH-US study participants. The CVAC includes five themes for each county in the U.S: sociodemographic barriers (i.e., socioeconomic disadvantage, lack of access to information), limited healthcare system resources (i.e., healthcare system capacity, healthcare quality, health spending per capita and total healthcare funding per capita), healthcare accessibility barriers (i.e., cost barriers, transportation barriers), irregular care-seeking behavior (i.e., lack of a designated medical home, lack of routine care visits) and historic undervaccination (i.e., lower coverage and high refusal rates of various vaccines). Historic undervaccination is hereafter referred to as “history of low vaccination”.

The CVAC includes a score for the composite measure of all themes as well as scores for the individual themes. Missing data values were imputed with median values across all counties. Scores were calculated using stepwise percentile ranking and equal weighting of the themes for the composite measure and subthemes for the individual theme measures (16). Scores for the composite measure (i.e., “overall county-level vaccination barriers”), as well as the individual theme measures (i.e., “component measures”) ranged from 0 to 1, with higher scores representing greater barriers.

Consistent with the original coding scheme outlined in the CVAC Methodology report (16), the composite measure and the component measures were categorized as “low/medium” (i.e., counties with a barrier score ≤ 0.6), “high” (i.e., counties with a barrier score >0.6 and ≤ 0.8), and “very high” (i.e., counties with a barrier score >0.8).

#### Willingness to receive the COVID-19 vaccine

Willingness to receive the COVID-19 vaccine was measured using the item “Do you plan to get the COVID-19 vaccine once it becomes available?”. Response options included “Definitely not,” “Probably not,” “Probably yes,” “Definitely yes,” “I have received one dose of the COVID-19 vaccine,” and “I have received two doses of the COVID-19 vaccine.” Willingness to receive the COVID-19 vaccine was coded with “Definitely not” coded as 1, “Probably not” coded as 2, “Probably yes” coded as 3, and “Definitely yes,” “I have received one dose of the COVID-19 vaccine,” and “I have received two doses of the COVID-19 vaccine” coded as 4.

#### Racial/ethnic group and sociodemographic covariates

Participants self-identified whether they were of Hispanic, Latino, or Spanish origin (i.e., “No” or “Yes”). In addition, those who responded that they were of Hispanic, Latino, or Spanish origin, were asked to select their language preference for the survey (i.e., English or Spanish). Lastly, participants self-identified their race from the categories used by the 2020 U.S. Census. Participants who selected “Yes” to the item capturing Hispanic, Latino, or Spanish origin were included in the subgroup labeled “Hispanic/Latino” which included Hispanic/Latino participants from all gender identities. The Hispanic/Latino ethnicity, language preference, and race items were combined to create categories of American Indian/Alaska Native, Asian, Black/African American, Hispanic/Latino-English Language Preference (ELP), Hispanic/Latino-Spanish Language Preference (SLP), Multiracial, Native Hawaiian/Pacific Islander, and White.

Sociodemographic characteristics used as covariates in the analysis included age in years (i.e., 18–34, 35–49, 50–64, and 65 and older), gender (i.e., man, woman, non-binary, transgender, not listed), education level (i.e., high school or less, some college or 2-year college, 4-year college, and post-graduate), and annual household income (i.e., <$20,000, $20,000–49,999, $50,000–99,999, and $100,000 and over). Additionally, political ideology (i.e., conservative, liberal, moderate, not sure) and health insurance coverage (i.e., covered or not covered) were included as covariates given their previously established associations with COVID-19 vaccination intentions (21, 22). Lastly, at the time of data collection, only adults with high-risk chronic health conditions were eligible to receive the COVID-19 vaccine. Given this eligibility, participants with high-risk chronic health conditions may have been more likely to have received at least one dose of the COVID-19 vaccine. Therefore, the presence of a high-risk chronic health condition was included as a covariate in the analysis. Participants reported whether they had a chronic health condition by indicating whether a medical doctor or health professional ever told them they had a chronic health condition (i.e., checking all that apply from a list of chronic health conditions). Participants' responses were coded as “high-risk” (1) or “not high-risk” (0) using the CDC guidelines on medical conditions that increased the risk for severe illness due to COVID-19 (23).

### Data analysis

Descriptive statistics and chi-square difference tests were conducted in R version 4.2.1. The proportions of sociodemographic characteristics and county-level vaccination barriers were assessed in the total population and stratified by racial/ethnic groups. Willingness to receive the COVID-19 vaccine was assessed in total population and stratified by county-level vaccination barriers. Multigroup regression analyses were conducted in M*plus* version 8.6 (24). Ordinal logistic regression models included the maximum likelihood with robust standard errors estimator (MLR). Given the use of the MLR estimator, the Satorra-Bentler scaled chi-square test for model comparisons was calculated using the SBSDiff package in R (25). All adjusted models included race/ethnicity (except for multigroup models in which race/ethnicity was used as a stratification variable), age, gender, annual household income, education level, political ideology, health insurance, and high-risk chronic health condition. All analyses were weighted to be nationally representative within each racial/ethnic group.

#### Racial/ethnic differences in county-level vaccination barriers

Racial/ethnic differences in the prevalence of county-level vaccination barriers (i.e., separate models for the composite measure and each of the component measures) were estimated using unadjusted and adjusted ordinal logistic regression. County-level vaccination barriers were treated as an ordinal variable, therefore, odds ratios >1 indicated whether each racial/ethnic group was more likely to live in a county with higher barriers (i.e., higher categories vs. lower categories; “very high” vs. “high” and “low/medium”; “very high” and “high” vs. “low/medium”) compared to White adults.

#### County-level vaccination barriers associated with willingness to receive the COVID-19 vaccine

The association between county-level vaccination barriers and willingness to receive the COVID-19 vaccine was estimated using unadjusted and adjusted ordinal logistic regression. Odds ratios <1 indicated that counties with high (vs. low/medium) and very high (vs. low/medium) county-level vaccination barriers were less willing to receive the COVID-19 vaccine.

#### Racial/ethnic differences in associations between county-level vaccination barriers and willingness to receive the COVID-19 vaccine

Multigroup structural equation modeling was used to examine racial/ethnic group differences in the magnitude of the associations between county-level vaccination barriers and willingness to receive the COVID-19 vaccine. Given the small sample cell sizes of specific gender categories (i.e., non-binary, transgender, and not listed), ridge estimators were used to address singularity issues (i.e., lack of variance due to small cell sizes within racial/ethnic groups). The ridge estimator included a small constant value added to the diagonal of the covariance matrix (26, 27) and allowed for inclusion of all gender categories in the analysis. When gender categories were excluded from the analysis without the use of ridge estimators, results were consistent (data available upon request), therefore the model including all gender categories was used for the final analysis.

All models were estimated using unadjusted and adjusted ordinal logistic regression. Fully constrained models (i.e., associations between the county-level vaccination barriers and willingness to receive the COVID-19 vaccine held equal across racial/ethnic groups) were compared to freely estimated models (i.e., associations between the county-level vaccination barriers and willingness to receive the COVID-19 vaccine allowed to vary freely across racial/ethnic groups). Model comparisons were used to assess whether allowing each association to vary freely across racial/ethnic groups resulted in a stronger model fit (i.e., a significant difference in the log-likelihood values of the two models indicated significant interactions between the county-level vaccination barrier and race/ethnicity in these models). Model comparisons that revealed significant racial/ethnic differences were further assessed by examining the race/ethnicity-stratified results of the freely estimated multigroup model.

## Results

Participant sociodemographic characteristics in the total population and stratified by race/ethnicity are reported in Table 1 (unweighted estimates presented in Supplementary Table S1).

**Table 1:** Sociodemographic characteristics of study population, total population and stratified by race/ethnicity.

	**Total (*N =* 5,479)**	**AI/AN^a^ (*n =* 498)**	**Asian (*n =* 997)**	**Black/AA^b^ (*n =* 995)**	**Hispanic/ Latino ELP^c^ (*n =* 495)**	**Hispanic/ Latino SLP^d^ (*n =* 503)**	**Multiracial (*n =* 499)**	**NH/PI^e^ (*N =* 499)**	**White (*N =* 992)**
**Age (years), %**									
18–34	34.7	35.0	33.9	33.4	42.3	35.6	47.2	36.7	25.2
35–49	26.8	23.0	29.6	23.9	26.7	36.3	24.8	34.4	21.0
50–64	23.8	26.3	23.1	26.8	13.4	24.6	17.9	22.0	29.0
65 and older	14.7	15.7	13.4	15.9	17.6	3.5	10.1	6.9	24.8
**Gender, %**									
Man	43.9	35.3	45.5	46.4	49.2	38.5	45.9	33.6	48.1
Woman	54.2	61.9	53.1	53.0	49.7	61.1	47.3	63.4	50.5
Non-binary	1.1	2.4	0.6	0.5	0.6	0.2	4.5	0.3	0.8
Transgender	0.3	0.3	0.2	0.1	0.2	0.2	0.3	2.2	0.0
Not listed	0.5	0.1	0.6	0.0	0.3	0.0	2.0	0.5	0.6
**Education, %**									
High school or less	39.8	42.6	23.7	42.2	53.2	67.2	32.1	45.8	32.5
Some college, 2-year college	33.0	44.6	23.7	38.1	31.1	21.0	38.0	37.9	33.3
4-year college	16.6	7.7	30.5	12.0	8.7	9.1	18.8	11.6	21.1
Post graduate	10.6	5.1	22.1	7.7	7.0	2.7	11.1	4.7	13.1
**Income, %**									
Less than $20 K	29.6	42.7	18.2	41.2	29.4	32.0	24.6	37.9	20.3
$20–49 K	30.9	29.8	25.8	30.3	35.5	48.7	27.8	26.6	29.8
$50–100 K	25.1	20.2	28.6	19.5	24.8	15.3	33.0	26.0	30.3
$100 K and over	14.4	7.3	27.4	9.0	10.3	4.0	14.6	9.5	19.6
**Political ideology, %**									
Conservative	23.7	29.0	19.0	15.1	21.7	17.9	16.1	26.7	40.5
Liberal	29.6	22.7	32.8	31.7	33.8	26.4	42.5	19.2	26.0
Moderate	32.6	30.1	39.3	37.0	31.8	28.2	31.5	30.6	26.8
Not sure	14.1	18.2	8.9	16.2	12.7	27.5	9.9	23.5	6.7
**Health insurance coverage, %**									
Covered	84.8	87.8	91.0	84.4	82.2	58.4	88.7	83.6	90.8
Not covered	15.2	12.2	9.0	15.6	17.8	41.6	11.3	16.4	9.2
**High-risk chronic health condition, %**									
One or more high-risk chronic health conditions	41.9	50.5	33.3	48.4	37.4	31.1	41.4	41.8	47.6
No high-risk chronic health conditions	58.1	49.5	66.7	51.6	62.6	68.9	58.6	58.2	52.4

Weighted to be nationally representative within each racial/ethnic group.

a AI/AN = American Indian/Alaska Native.

b B/AA = Black/African American.

c English Language Preference.

d Spanish Language Preference.

e NH/PI = Native Hawaiian/Pacific Islander.

All χ2 test *p*-values < 0.05.

### Racial/ethnic differences in county-level vaccination barriers

In the total study population, most participants lived in counties with low or medium overall county-level vaccination barriers (64.0%), sociodemographic barriers (80.2%), limited healthcare system resources (76.0%), healthcare accessibility barriers (66.7%), and history of low vaccination (52.1%) (Table 2). Almost half of participants lived in areas with low or medium irregular care-seeking behavior (45.3%). These proportions varied across racial/ethnic groups (global chi-square difference tests and pairwise comparisons revealed significant differences in county-level vaccination barriers between specific groups (*p*-values < 0.01)). For example, the proportions of adults living in counties with high or very high overall county-level vaccination barriers and sociodemographic barriers were higher for American Indian/Alaska Native (25.4% and 15.9%), Black/African American (25.0% and 12.0%), and Hispanic/Latino adults (ELP: 30.5% and 23.0%; SLP: 36.6% and 24.9%%) and lower for Asian (21.0% and 5.7%), Native Hawaiian/Pacific Islander (17.5% and 10.5%), and White adults (18.8% and 6.5%). Higher proportions of Native Hawaiian/Pacific Islander adults lived in counties with a very high history of low vaccination (42.5%) and high or very high irregular care-seeking behaviors (49.0% and 29.8%).

**Table 2:** Prevalence of county-level vaccination barriers, measured using the COVID-19 vaccine coverage index (CVAC) and willingness to receive the COVID-19 vaccine, total population and stratified by race/ethnicity.

	**Total (*N =* 5,479)**	**AI/AN^a^ (*n =* 498)**	**Asian (*n =* 997)**	**Black/AA^b^ (*n =* 995)**	**Hispanic/ Latino ELP^c^ (*n =* 495)**	**Hispanic/ Latino SLP^d^ (*n =* 503)**	**Multiracial (*n =* 499)**	**NH/PI^e^ (*N =* 499)**	**White (*N =* 992)**
**Overall county-level vaccination barriers, %**									
Low/medium	64.0	58.7	73.3	63.0	46.5	38.5	65.9	72.0	74.7
High	24.1	25.4	21.0	25.0	30.5	36.6	25.6	17.5	18.8
Very high	11.9	15.9	5.7	12.0	23.0	24.9	8.5	10.5	6.5
**Sociodemographic barriers, %**									
Low/medium	80.2	72.1	87.9	74.6	74.5	73.9	83.1	85.1	84.1
High	12.7	14.6	10.2	14.3	15.5	15.3	11.9	11.8	10.8
Very high	7.1	13.3	1.9	11.1	10.0	10.8	5.0	3.1	5.1
**Limited healthcare system resources, %**									
Low/medium	76.0	71.9	85.5	75.4	72.1	62.5	76.5	83.1	74.2
High	14.7	15.3	9.2	16.7	17.3	24.6	14.4	6.8	15.6
Very high	9.3	12.8	5.3	7.9	10.6	12.9	9.1	10.1	10.2
**Healthcare accessibility barriers, %**									
Low/medium	66.7	64.1	77.8	49.8	62.3	54.2	68.3	82.5	73.8
High	20.0	18.7	16.2	26.4	17.8	26.4	21.6	13.4	18.8
Very high	13.2	17.2	6.0	23.8	19.9	19.4	10.1	4.1	7.4
**Irregular care-seeking behavior, %**									
Low/medium	45.3	36.4	44.3	55.6	27.7	23.0	50.4	41.9	59.7
High	19.4	26.6	15.3	23.1	17.7	18.0	19.5	15.6	19.3
Very high	35.3	37.0	40.4	21.3	54.6	59.0	30.1	42.5	21.0
**History of low vaccination, %**									
Low/medium	52.1	45.5	48.8	70.3	41.7	43.9	52.6	21.2	65.1
High	21.0	26.6	24.2	11.9	19.4	17.6	19.5	49.0	13.4
Very high	26.9	27.9	27.0	17.8	38.9	38.5	27.9	29.8	21.5

Weighted to be nationally representative within each racial/ethnic group.

a AI/AN=American Indian/Alaska Native.

b B/AA=Black/African American.

c English Language Preference.

d Spanish Language Preference.

e NH/PI=Native Hawaiian/Pacific Islander.

All χ2 test *p*-values < 0.05

**Figure 1:**
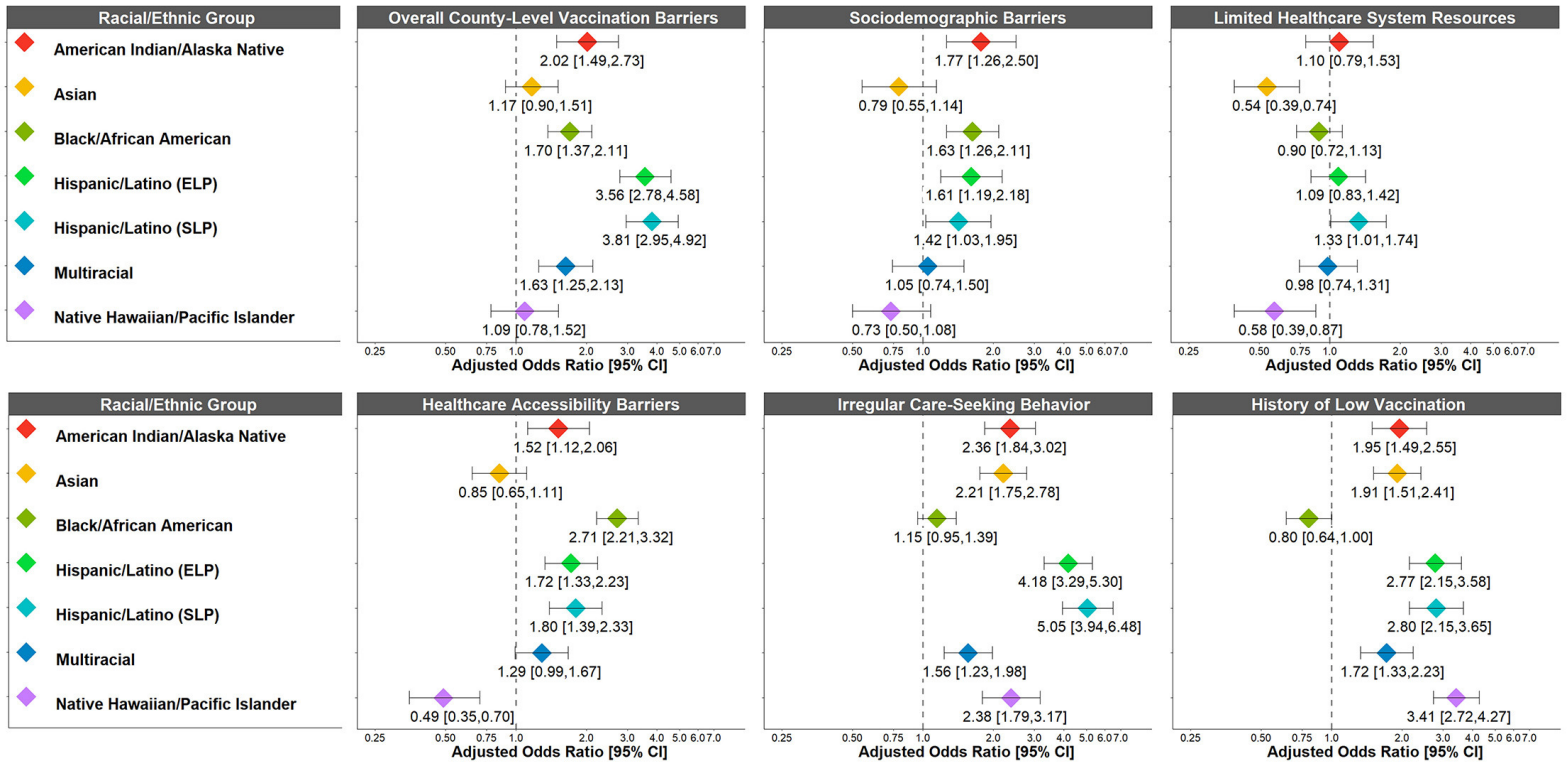
Racial/ethnic differences in odds of living in a county with higher COVID-19 vaccination barriers (“very high” vs. “high” and “low/medium”; “very high” and “high” vs. “low/medium”). COVID-19 vaccination barriers were treated as an ordinal variable. Adjusted odds ratios >1 indicate that the racial/ethnic group was more likely to live in a county with higher COVID-19 vaccination barriers compared to White adults (reference group). Weighted to be nationally representative within each racial/ethnic group. Adjusted for race/ethnicity, age, gender, annual household income, education level, political ideology, health insurance coverage, and high-risk chronic health condition. ELP, English Language Preference; SLP, Spanish Language Preference.

After adjustment, American Indian/Alaska Native (adjusted odds ratio (AOR): 2.02, 95% CI: 1.49–2.73), Black/African American (AOR: 1.70, 95% CI: 1.37–2.11), Hispanic/Latino (ELP AOR: 3.56, 95% CI: 2.78–4.58; SLP AOR: 3.81, 95% CI: 2.95–4.92), and Multiracial (AOR: 1.63, 95% CI: 1.25–2.13) adults were more likely than White adults to live in counties with higher overall county-level vaccination barriers (Figure 1). No differences were observed between Asian, Native Hawaiian/Pacific Islander, and White adults.

However, the patterns of racial/ethnic differences varied across the component barriers (Figure 1). American Indian/Alaska Native (AOR: 1.77, 95% CI: 1.26–2.50), Black/African American (AOR: 1.63, 95% CI: 1.26–2.11), and Hispanic/Latino (ELP AOR: 1.61, 95% CI: 1.19–2.18; SLP AOR: 1.42, 95% CI: 1.03–1.95) adults were more likely than White adults to live in counties with higher sociodemographic barriers. Similarly, American Indian/Alaska Native (AOR: 1.12, 95% CI: 1.52–2.06), Black/African American (2.71, 95% CI: 2.21–3.32), Hispanic/Latino ELP (AOR: 1.72, 95% CI: 1.33–2.23) and SLP Hispanic/Latino (AOR: 1.80, 95% CI: 1.39–2.33), and Multiracial (AOR: 1.29, 95% CI: 0.99–1.67) adults were more likely than White adults to live in counties with higher healthcare accessibility barriers. By contrast, Asian and Native Hawaiian/Pacific Islander adults were less likely than White adults to live in counties with higher sociodemographic barriers, limited healthcare system resources, and healthcare accessibility barriers (although the confidence intervals for some of these associations were wide). All marginalized racial/ethnic groups were more likely than White adults to live in counties with higher irregular care-seeking behavior (AORs: 1.15–5.05). This trend was similar for history of low vaccination (AORs: 1.72–3.41), except for Black/African American adults who were less likely than White adults to live in counties with higher history of low vaccination (AOR: 0.80, 95% CI: 0.64–1.00).

### County-level vaccination barriers associated with willingness to receive the COVID-19 vaccine

In the total study population, most participants were willing to vaccinate [“Definitely yes/received ≥1 dose”: 40.1%; or “Probably yes”: 27.0%)], but COVID-19 vaccination intentions varied across county-level vaccination barriers (Table 3). In counties with low/medium barriers, the proportions of those who reported “Definitely yes/received ≥1 dose” were roughly 42%, but these proportions were lower in counties with high or very high barriers (e.g., 31.9% in counties with high sociodemographic barriers, 34.2% in counties with very high healthcare accessibility barriers). These trends were similar across all barriers, except for history of low vaccination, in which willingness to receive the COVID-19 vaccine did not significantly vary across levels of this barrier (*p*-value = 0.29).

**Table 3:** County-level vaccination barriers stratified by COVID-19 vaccination intentions.

	**Definitely not (*n =* 896)**	**Probably not (*n =* 906)**	**Probably yes (*n =* 1,482)**	**Definitely yes/received ≥1 dose (*n =* 2,195)**	***p*-value**
**Overall, %**	**16.4**	**16.5**	**27.0**	**40.1**	
**Overall county-level vaccination barriers, %**					< 0.01
Low/medium	15.9	15.9	26.5	41.7	
High	16.4	18.6	26.3	38.7	
Very high	19.0	15.6	31.3	34.1	
**Sociodemographic barriers, %**					< 0.01
Low/medium	15.2	16.4	26.6	41.8	
High	22.0	16.8	29.3	31.9	
Very high	19.8	17.6	27.6	34.9	
**Limited healthcare system resources, %**					< 0.01
Low/medium	15.6	15.8	27.0	41.6	
High	19.7	18.5	26.5	35.3	
Very high	16.9	19.6	28.3	35.1	
**Healthcare accessibility barriers, %**					< 0.01
Low/medium	15.2	16.2	26.4	42.1	
High	18.8	17.9	26.2	37.1	
Very high	18.3	16.0	31.5	34.2	
**Irregular care-seeking behavior, %**					< 0.01
Low/medium	17.3	17.2	27.0	38.5	
High	18.6	17.0	24.3	40.0	
Very high	13.9	15.4	28.6	42.1	
**History of low vaccination, %**					0.29
Low/medium	16.8	16.3	26.7	40.2	
High	14.0	17.2	28.8	40.1	
Very high	17.3	16.4	26.4	39.9	

Weighted to be nationally representative within each racial/ethnic group.

Global chi-square difference tests used to generate *p*-values.

In the unadjusted models, participants who lived in counties with very high overall county-level vaccination barriers (vs. low/medium), were less willing to receive the COVID-19 vaccine (OR: 0.79, 95% CI: 0.66–0.94) (Supplementary Table S2). In addition, compared to low/medium barriers, those who lived in counties with both high and very high sociodemographic barriers (high OR: 0.67, 95% CI: 0.56–0.81; very high OR: 0.75, 95% CI: 0.60–0.94), limited healthcare system resources (high OR: 0.76, 95% CI: 0.64–0.89; very high OR: 0.80, 95% CI: 0.66–0.97) and healthcare accessibility barriers (high OR: 0.80, 95% CI: 0.69–0.92; very high OR: 0.78, 95% CI: 0.67–0.92) were less willing to receive the COVID-19 vaccine. Participants who lived in counties with very high irregular care-seeking behavior (vs. low/medium) were more willing to receive the COVID-19 vaccine (OR: 1.21, 95% CI: 1.07–1.38)

However, after adjustment, these results were largely attenuated and no longer significantly associated with willingness to receive the COVID-19 vaccine, except for sociodemographic barriers and irregular care-seeking behavior (Table 4). Those who lived in counties with high sociodemographic barriers (vs. low/medium) were still less willing to receive the COVID-19 vaccine (AOR: 0.78, 95% CI: 0.64–0.94) and those who lived in counties with very high irregular care-seeking behavior (vs. low/medium) were still more willing to receive the COVID-19 vaccine (AOR: 1.20, 95% CI: 1.04–1.39) after adjustment.

**Table 4:** Associations between county-level vaccination barriers and willingness to receive the COVID-19 vaccine.

	**High vs. low/medium barriers**		**Very high vs. low/medium barriers**	
**County-level barrier**	**AOR**	**95% CI**	**AOR**	**95% CI**
Overall county-level vaccination barriers	1.03	0.88–1.12	0.94	0.78–1.14
Sociodemographic barriers	0.78	0.64–0.94	1.05	0.82–1.35
Limited healthcare system resources	0.86	0.72–1.02	0.94	0.77–1.15
Healthcare accessibility barriers	0.92	0.79–1.06	1.03	0.86–1.23
Irregular care-seeking behavior	1.09	0.92–1.29	1.20	1.04–1.39
History of low vaccination	1.05	0.89–1.24	0.95	0.81–1.11

Weighted to be nationally representative within each racial/ethnic group.

Adjusted odds ratios (AOR) and 95% confidence intervals (95% CI) of willingness to receive the COVID-19 vaccine.

AOR < 1 indicates that counties with high or very high (vs. low/medium) county-level vaccination barriers were less willing to receive the COVID-19 vaccine.

Adjusted for race/ethnicity, age, gender, annual household income, education level, political ideology, health insurance coverage, and high-risk chronic health condition.

### Racial/ethnic differences in the association between county-level vaccination barriers and willingness to receive the COVID-19 vaccine

**Figure 2:**
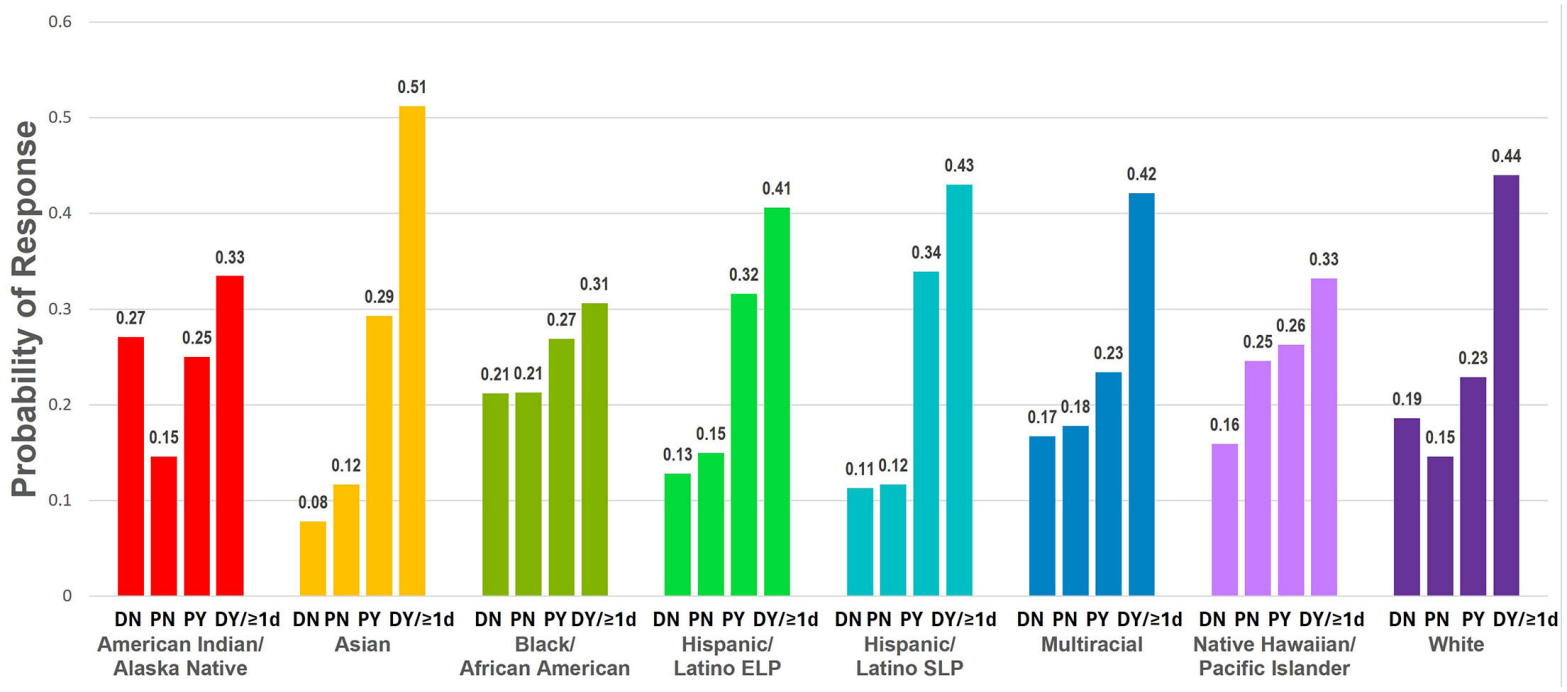
Racial/ethnic differences in predicted probabilities of willingness to receive the COVID-19 vaccine. Adjusted predicted probabilities of willingness to receive the COVID-19 vaccine for each racial/ethnic group (DN, Definitely not; PN, Probably not; PY, Probably yes; DY/≥1d, Definitely yes, already received ≥1 dose of the COVID-19 vaccine). Weighted to be nationally representative within each racial/ethnic group. Adjusted for age, gender, annual household income, education level, health insurance, high-risk chronic health condition, and political ideology across racial/ethnic groups. ELP, English Language Preference; SLP, Spanish Language Preference.

The multigroup analysis allowed for examining racial/ethnic differences in the predicted probabilities of willingness to receive the COVID-19 vaccine, adjusting for age, gender, annual household income, education level, political ideology, health insurance, and high-risk chronic health condition (Figure 2).

Multigroup comparisons revealed significant racial/ethnic differences in the magnitude of association between history of low vaccination and willingness to receive the COVID-19 vaccine (*p*-value = 0.01) and marginally significant differences in the magnitude of association between overall county-level vaccination barriers and willingness to receive the COVID-19 vaccine (*p*-value = 0.09) (Supplementary Table S3). Apart from these differences, there were no significant differences between the fully constrained and freely estimated models for the remaining component barriers (i.e., indicating relatively consistent associations between barriers and vaccination intentions across racial/ethnic groups).

**Figure 3:**
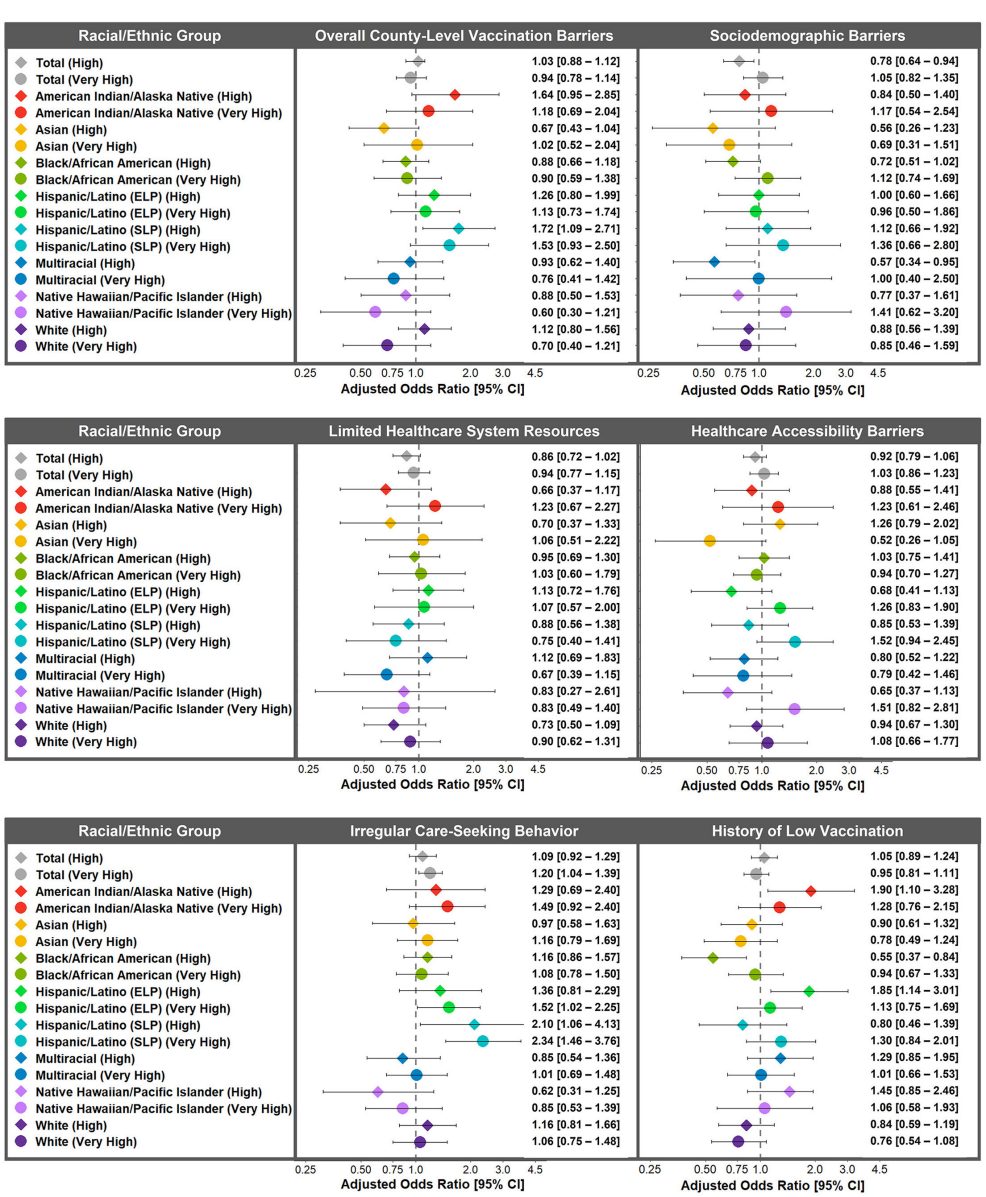
Racial/ethnic differences in adjusted associations between county-level vaccination barriers and willingness to receive the COVID-19 vaccine. Adjusted odds ratios of willingness to receive the COVID-19 vaccine for high and very high (vs. low/medium) county-level vaccination barriers for the overall study population and within each racial/ethnic group. AOR <1 indicates that counties with high or very high (vs. low/medium) county-level vaccination barriers were less willing to receive the COVID-19 vaccine. Weighted to be nationally representative within each racial/ethnic group. Adjusted for age, gender, annual household income, education level, health insurance, high-risk chronic health condition, and political ideology across racial/ethnic groups. ELP, English Language Preference; SLP, Spanish Language Preference.

Trends in the magnitudes of association for overall county-level vaccination barriers and history of low vaccination were examined in the multigroup models (Figure 3). Although confidence intervals were wide, the results revealed different patterns in the associations across racial/ethnic groups. American Indian/Alaska Native adults (high AOR: 1.64, 95% CI: 0.95–2.85; very high AOR: 1.18, 95% CI: 0.69–2.04) and Hispanic/Latino ELP adults (high AOR: 1.26, 95% CI: 0.80–1.99; very high AOR: 1.13, 95% CI: 0.73–1.74) and Hispanic/Latino SLP adults (high AOR: 1.72, 95% CI: 1.09–2.71; very high AOR: 1.53, 95% CI: 0.93–2.50) who lived in counties with high or very high overall county-level vaccination barriers (vs. low/medium) were more willing to receive the COVID-19 vaccine. Alternatively, Asian adults (high AOR: 0.67, 95% CI: 0.43–1.04), Black/African American adults (high AOR: 0.88, 95% CI: 0.66–1.18; very high AOR: 0.90, 95% CI: 0.59–1.38), Multiracial adults (high AOR: 0.93, 95% CI: 0.62–1.40; very high AOR: 0.76, 95% CI: 0.41–1.42), and Native Hawaiian/Pacific Islander adults (high AOR: 0.88, 95% CI: 0.50–1.53; very high AOR: 0.60, 95% CI: 0.30–1.21) who lived in counties with high or very high (vs. low/medium) overall county-level vaccination barriers were less willing to receive the COVID-19 vaccine.

In assessing racial/ethnic differences in the association between history of low vaccination and COVID-19 vaccination intentions, American Indian/Alaska Native adults (high AOR: 1.90, 95% CI: 1.10–3.28) and Hispanic/Latino ELP adults (high AOR: 1.85, 95% CI: 1.14–3.01) living in counties with a high history of low vaccination (vs. low/medium) were more willing to receive the COVID-19 vaccine (Figure 3). Interestingly, Black/African American adults who lived in counties with a high history of low vaccination were less willing to receive the COVID-19 vaccine (AOR: 0.55, 95% CI: 0.37–0.84), yet the association was weaker and non-significant for Black/African American adults who lived in counties with a very high history of low vaccination (AOR: 0.94, 95% CI: 0.67–1.33).

No racial/ethnic differences in the associations between sociodemographic barriers, limited healthcare system resources, healthcare accessibility barriers, or irregular care-seeking behaviors were observed after adjustment.

## Discussion

This study used the newly developed CVAC to examine whether county-level vaccination barriers varied across racial/ethnic groups in the U.S. and were associated with willingness to receive the COVID-19 vaccine. In addition, this study assessed whether these associations differed across racial/ethnic groups. Importantly, this study was conducted using a large survey of U.S. adults across seven racial/ethnic groups, including substantial representation from American Indian/Alaska Native, Native Hawaiian/Pacific Islander, and Multiracial adults who are often underrepresented in national surveys. In the total study population, higher county-level vaccination barriers were generally associated with less willingness to receive the COVID-19 vaccine, yet many of the associations were largely attenuated after adjusting for covariates. The trends in the associations between county-level vaccination barriers and willingness to receive the COVID-19 vaccine varied by type of barrier and, in some cases, varied across racial/ethnic groups.

Consistent with previous studies on racial/ethnic disparities by geographic region (28, 29), American Indian/Alaska Native, Black/African American, Hispanic/Latino ELP and SLP, and Multiracial adults were more likely than White adults to live in counties with higher overall county-level vaccination barriers and multiple component barriers. Trends for Asian and Native Hawaiian/Pacific Islander adults varied across component barriers. Asian and Native Hawaiian/Pacific Islander adults were more likely than White adults to live in counties with higher histories of low vaccination and irregular care-seeking behavior, but less likely than White adults to live in counties with higher sociodemographic barriers, limited healthcare system resources and healthcare accessibility barriers. The lower odds of Asian and Native Hawaiian/Pacific Islander adults living in counties with limited healthcare system resources and higher healthcare accessibility barriers may relate to disparities in rural vs. urban healthcare. For example, there is existing evidence that higher proportions of Asian and Native Hawaiian/Pacific Islander adults live in urban areas which tend to score higher on healthcare system quality and access than rural areas (30, 31). Moreover, the higher odds of Asian and Native Hawaiian/Pacific Islander adults living in counties with histories of low vaccination and irregular care-seeking behavior may relate to previous evidence that Asian and Native Hawaiian/Pacific Islander adults were less likely to have a usual primary care provider compared with White adults (32). Together, racial/ethnic differences in county-level vaccination barriers suggest that healthcare system resources and access to healthcare are inequitably distributed across counties in the U.S. Furthermore, counties with greater healthcare barriers are often those with higher proportions of residents from marginalized racial/ethnic groups.

Moreover, participants who lived in counties with greater overall county-level vaccination barriers and greater component barriers (with the exception of history of low vaccination) were less willing to receive the COVID-19 vaccine, which is consistent with prior analyses using the CVAC (33). After adjusting for individual-level sociodemographic covariates, however, overall county-level vaccination barriers, limited healthcare system resources, and healthcare accessibility barriers were no longer significantly associated with willingness to receive the COVID-19 vaccine. These findings suggest that individual-level factors may have affected the association between these county-level vaccination barriers and willingness to receive the COVID-19 vaccine by serving as confounders and/or mediators in these relationships. The influence of the individual-level sociodemographic covariates on willingness to receive the COVID-19 vaccine is consistent with previous literature (22, 34–36) and further suggests that many sociodemographic characteristics associated with COVID-19 vaccination intentions vary systematically across counties in the U.S.

Despite the impact of individual-level sociodemographic covariates on willingness to receive the COVID-19 vaccine, participants who lived in counties with higher sociodemographic barriers (i.e., county-level measures of socioeconomic disadvantage and lack of access to information) were still less willing to receive the COVID-19 vaccine after adjustment. These findings suggest that even after adjusting for individual-level sociodemographic characteristics, sociodemographic barriers at the county-level can affect individuals' willingness to receive the COVID-19 vaccine. In addition, irregular care-seeking behavior was also associated with willingness to receive the COVID-19 vaccine after adjustment. In contrast, participants who lived in counties with higher irregular care-seeking behavior were more willing to receive the COVID-19 vaccine. There may be multiple explanations for this finding. One possibility is that the measure of irregular care-seeking behavior partially captured relatively healthy individuals who required less routine care due to their relatively healthy status. Yet, these individuals may have been more willing to receive recommended vaccinations as found in previous research [e.g., self-rated health among U.S. adults was associated with lower likelihood of having a usual source of care, but also a greater willingness to receive recommended vaccinations (37, 38)]. Given this possibility, it may be important to consider whether irregular care-seeking behavior is an appropriate measure to include in the CVAC and/or whether it is possible to capture irregular care-seeking behavior related to delays in necessary care.

There were relatively few racial/ethnic differences in the associations between county-level vaccination barriers and willingness to receive the COVID-19 vaccine. These findings may suggest a generally consistent relationship between most CVAC county-level vaccination barriers and willingness to receive a COVID-19 vaccine across racial/ethnic groups. However, some notable differences included that Black/African American adults who lived in counties with a higher history of low vaccination were less willing to receive the COVID-19 vaccine and this association was significant after adjusting for sociodemographic covariates. These findings are consistent with previous studies in which both influenza vaccination and COVID-19 vaccination were lowest among Black/African American adults (39). Furthermore, these findings may suggest that factors preventing healthcare system use among Black/African American individuals prior to the COVID-19 pandemic (e.g., disparities in care, anticipated healthcare discrimination) likely contributed to similar trends in COVID-19 vaccination disparities.

In contrast, in some racial/ethnic groups, higher county-level vaccination barriers were associated with greater willingness to receive the COVID-19 vaccine. Compared to low/medium barriers, Hispanic/Latino SLP adults who lived in counties with high overall county-level vaccination barriers and Hispanic/Latino ELP adults and American Indian/Alaska Native adults who lived in counties with high histories of low vaccination were more willing to receive the COVID-19 vaccine. Given prior findings that many of the factors associated with county-level vaccination barriers in the U.S. (e.g., lack of internet access and/or limited healthcare system resources) were also associated with lower income (40), it is possible that income disparities across counties were driving these findings. Individuals in counties with greater vaccination barriers due to lower county-level income, may have perceived greater COVID-19 risks due to economic factors (e.g., living and working conditions that increased exposure to COVID-19). These concerns may have increased their willingness to receive the COVID-19 vaccine. This potential explanation is further supported by previous evidence that Hispanic/Latino and American Indian/Alaska Native adults perceived higher COVID-19 risk compared to other racial/ethnic groups (39) and that Hispanic/Latino adults from households with lower income had greater worries about contracting COVID-19 (41).

Public health interventions might partially explain why higher county-level vaccination barriers were associated with greater willingness to receive the COVID-19 vaccine among Hispanic/Latino and American Indian/Alaska Native adults. In addition, public health efforts might also explain why a high history of low vaccination was associated with less willingness to receive the COVID-19 vaccine among Black/African American adults, but not for a very high history of low vaccination. Many efforts to increase COVID-19 vaccination in Hispanic/Latino, American Indian/Alaska Native, and Black/African American communities began prior to the release of the COVID-19 vaccine (42–44). Moreover, many of these interventions focused on areas at high risk of reporting COVID-19 vaccine hesitancy. Given that the REACH-US study was conducted from January 2021 to March 2021, it is possible that the survey captured COVID-19 vaccination intentions that were positively influenced by these health communication campaigns. This potential explanation would demonstrate the benefit of targeted outreach efforts in increasing willingness to receive the COVID-19 vaccine.

Several limitations should be considered when interpreting the present study findings. The sample was drawn from individuals who were not only online but also willing to participate in studies (45). Previous studies found that lack of internet access was a barrier to COVID-19 vaccination (46, 47), therefore COVID-19 vaccination intentions reported by the YouGov survey panel may be higher than vaccination intentions in the total U.S. population. In addition, the study was cross-sectional, which limits the ability to make causal inferences. Moreover, the present study assessed willingness to receive the COVID-19 vaccine, yet individuals' COVID-19 vaccination intentions may not have translated into their actual COVID-19 vaccination rates. It is possible, for example, that individuals overreported their intentions to receive the COVID-19 vaccine due to social desirability, as reported in recent studies (48). Alternatively, it is also possible that individuals who intended to get the COVID-19 vaccine later encountered barriers related to limited healthcare resources and accessibility. Given that self-reported COVID-19 vaccination intentions are often higher than actual rates of vaccination (49), the associations between many county-level vaccination barriers and COVID-19 vaccination uptake may be higher than the associations observed in the present study. Lastly, the present study used the CVAC which included some limitations in its development (16). The CVAC has yet to be fully validated and some counties with missing data required imputing data at the median county levels.

Despite these limitations, the present study had several strengths. The study included a large, diverse sample of U.S. adults with substantial representation of the racial/ethnic categories in the U.S. Census. Moreover, this study also considered language preference among Hispanic/Latino adults and consistent with previous studies (34), there were important differences between those who preferred to speak English and those who preferred to speak Spanish. This distinction reflects the heterogeneity of the U.S. Hispanic/Latino population on factors such as language preference (34, 50).

In addition, the present study focused on structural factors related to COVID-19 vaccination intentions, which have been underexplored in the literature. Studies that focus on structural barriers are particularly essential in public health interventions. Structural factors influence the distribution of public health interventions, including the ability to provide warnings and distribute treatment and protective actions within geographic areas and community structures (e.g., local businesses, schools, Federally Qualified Health Centers, religious organizations, and community-based organizations). Public health emergencies, such as responding to an infectious disease outbreak, require knowledge of the structural elements that may serve as barriers to plan accordingly for an efficient and timely response for all in the community.

Although willingness to receive the COVID-19 vaccine may have underestimated actual COVID-19 vaccination uptake, the measurement of COVID-19 vaccination intentions also represents a unique strength of this study. The willingness to receive the COVID-19 vaccine captured in the present study provides a snapshot of public perceptions of vaccination prior to its public release (51). Moreover, behavioral intentions are not only good indicators of subsequent behavior (52), but they represent thought processes that could highlight willingness to receive the COVID-19 vaccination in the absence of barriers. These findings, especially as they relate to county-level vaccination barriers may be helpful for responding to future pandemics.

There are many opportunities for future research related to the present study findings. Future studies could consider examining mediation pathways. It is possible that individual-level differences in sociodemographic characteristics mediate the relationships between county-level vaccination barriers and willingness to receive the COVID-19 vaccine. Limited healthcare system resources or healthcare accessibility barriers, for example, may have affected individuals' chronic health conditions and/or health insurance status, which in turn, influenced their willingness to receive the COVID-19 vaccine. These mediation pathways would be particularly important for public health efforts. Future studies might also consider examining more complex decision-making processes related to county-level vaccination barriers by using open-ended items and/or qualitative methods (e.g., interviews, focus groups) to explore COVID-19 vaccination intentions. In addition, future studies could examine whether the CVAC is a better predictor of actual COVID-19 vaccination rates compared to willingness to receive the COVID-19 vaccine. If willingness to receive the COVID-19 vaccine was higher than observed vaccination uptake rates, it might suggest that structural barriers prevented individuals from receiving the COVID-19 vaccine. Furthermore, if differences in willingness to receive the COVID-19 vaccine and observed COVID-19 vaccination rates varied across racial/ethnic groups, it could suggest the need to intervene at different stages across racial/ethnic groups (e.g., increasing awareness about COVID-19 vaccine benefits for groups with lower initial COVID-19 vaccination intentions, increasing COVID-19 vaccine access for those with high COVID-19 vaccination intentions, but lower observed rates). Lastly, the study of county-level factors associated with COVID-19 vaccination behavior raises awareness of contextual variables that could vary by county, as well as at the state and country level. Recent studies, for example, have examined how factors such as wealth, economic growth, population size, migration, and/or tourism impact COVID-19 death rates (53). These factors could be further examined as predictors of COVID-19 vaccination behavior.

The present study addressed recent calls to examine the importance of structural factors in COVID-19 vaccination rates (54–56). In examining COVID-19 vaccination intentions during the early stages of the COVID-19 vaccination program, the present study provides important information on how county-level vaccination barriers could have impacted initial COVID-19 vaccination intentions during the first year of the U.S. COVID-19 vaccination program. These findings have implications to assist in the planning of early-stage public health efforts for future vaccination interventions and may ultimately lead to infectious disease curtailment.

## Data availability statement

The raw data supporting the conclusions of this article will be made available by the authors, without undue reservation.

## Author contributions

JRF: conceptualization, methodology, software, formal analysis, data curation, visualization, writing—original draft, and writing—review and editing. PDS: formal analysis and writing—review and editing. JR and VMM: writing—review and editing. ATF: supervision, resources, funding acquisition, and writing—review and editing. All authors contributed to the article and approved the submitted version.
