# Supplementary material for: County-level barriers in the COVID-19 vaccine coverage index and their associations with willingness to receive the COVID-19 vaccine across racial/ethnic groups in the U.S
Source: Front Public Health. 2023 Oct 12;11:1192748. doi: 10.3389/fpubh.2023.1192748 (PMC10602638; doi:10.3389/fpubh.2023.1192748)
Supplement: Supplementary file 1 [file Table_1.pdf]

County-Level Barriers in the COVID-19 Vaccine Coverage Index and Willingness to Receive the COVID-19 Vaccine Across Racial/Ethnic Groups in the U.S. – Supplemental Material

Table S1. Sociodemographic characteristics of study population, total population and stratified by race/ethnicity (Unweighted, N=5475)

	Total (N=5475)	AI/AN[1] (n=498)	Asian (n=997)	Black/AA[2] (n=995)	Hispanic/ Latino ELP[3] (n=495)	Hispanic/ Latino SLP[4] (n=503)	Multiracial (n=499)	NH/PI[6] (N=499)	White (N=992)
**Age (Years), %**									
18-34	34.9	31.7	36.4	29.7	38.9	34.3	46.5	49.7	25.3
35-49	27.6	24.1	31.7	24.8	30.3	40.0	26.7	29.1	20.4
50-64	21.8	27.5	21.3	27.5	13.6	21.5	15.2	15.0	24.4
65 and older	15.7	16.7	10.6	18.0	17.2	4.2	11.6	6.2	29.9
**Gender, %**									
Man	42.3	37.4	47.5	42.8	43.3	32.3	41.1	32.3	48.2
Woman	55.8	60.4	51.1	56.6	55.1	64.9	52.1	64.9	50.3
Non-binary	1.1	1.8	0.8	0.5	0.6	0.8	4.6	0.8	0.9
Transgender	0.3	0.2	0.2	0.1	0.4	1.4	0.2	0.0	0.0
Not listed	0.5	0.2	0.4	0.0	0.6	0.6	2.0	0.6	0.6
**Education, %**									
High school or less	31.5	31.1	9.9	36.8	47.5	63.9	21.4	31.9	28.3
Some college, 2-year college	34.8	48.0	26.2	41.9	28.1	20.1	41.1	42.9	33.5
4-year college	20.0	12.5	36.7	12.9	10.4	10.8	24.5	15.8	23.7
Post graduate	13.7	8.4	27.2	8.4	14.0	5.2	13.0	9.4	14.5
**Income, %**									
Less than $20K	27.5	35.1	14.7	38.8	29.1	32.5	24.6	33.7	20.1
$20-49K	30.2	30.1	23.6	31.1	33.3	47.4	26.7	26.4	29.4
$50-100K	25.9	25.5	29.1	21.0	25.0	15.7	32.5	26.3	29.7
$100K and over	16.4	9.3	32.6	9.1	12.6	4.4	16.2	13.6	20.8
**Political Ideology, %**									
Conservative	21.2	28.9	15.9	14.0	22.8	18.1	16.0	22.7	32.4
Liberal	32.4	23.5	37.3	32.9	33.9	25.3	43.5	24.2	32.9
Moderate	33.4	33.5	39.9	37.4	30.7	28.7	30.3	33.1	28.3
Not sure	13.0	14.1	6.9	15.7	12.6	27.9	10.2	20.0	6.4
**Health Insurance Coverage, %**									
Covered	85.8	89.0	91.8	86.4	82.0	57.4	89.8	86.4	91.4
Not covered	14.2	11.0	8.2	13.6	18.0	42.6	10.2	13.6	8.6
**High Risk Chronic Health Condition, %**									
One or more high-risk chronic health conditions	41.6	53.4	30.0	50.7	36.1	31.1	41.9	38.3	47.9
No high-risk chronic health conditions	58.4	46.6	70.0	49.3	63.9	68.9	58.1	61.7	52.1

[1]AI/AN=American Indian/Alaska Native; [2]Asian; [3]Black/AA=African American; [4]Hispanic/Latino ELP=Hispanic/Latino English Language Preference; [5]Hispanic/Latino SLP=Hispanic/Latino Spanish Language Preference; [6]Multiracial; [7]NH/PI=Native Hawaiian/Pacific Islander; [8]White; All $\chi2$ test p-values <0.05
