# Supplementary material for: County-level barriers in the COVID-19 vaccine coverage index and their associations with willingness to receive the COVID-19 vaccine across racial/ethnic groups in the U.S
Source: Front Public Health. 2023 Oct 12;11:1192748. doi: 10.3389/fpubh.2023.1192748 (PMC10602638; doi:10.3389/fpubh.2023.1192748)
Supplement: Supplementary file 2 [file Table_2.pdf]

County-Level Barriers in the COVID-19 Vaccine Coverage Index and Willingness to Receive the COVID-19 Vaccine Across Racial/Ethnic Groups in the U.S. – Supplemental Material

Table S2. Unadjusted associations between county-level barriers & willingness to receive the COVID-19 vaccine

	High vs Low/Medium Barriers		Very High vs. Low/Medium Barriers	
**County-Level Barrier**	**OR**	**95% CI**	**OR**	**95% CI**
Overall County-Level Vaccination Barriers	0.89	0.78 – 1.02	0.79	0.66– 0.94
Sociodemographic Barriers	0.67	0.56 – 0.81	0.75	0.60 – 0.94
Limited Healthcare System Resources	0.76	0.64 – 0.89	0.80	0.66 – 0.97
Healthcare Accessibility Barriers	0.80	0.69 – 0.92	0.78	0.67 – 0.92
Irregular Care-Seeking Behavior	1.00	0.85 – 1.18	1.21	1.07 – 1.38
History of Low Vaccination	1.06	0.92 – 1.22	0.98	0.85 – 1.13

Weighted to be nationally representative within each racial/ethnic group

Odds ratios (OR) and 95% confidence intervals (95% CI) of willingness to receive the COVID-19 vaccine

OR<1 indicates that counties with high or very high (versus low/medium) county-level vaccination barriers were less willing to receive the COVID-19 vaccine
