# Supplementary material for: County-level barriers in the COVID-19 vaccine coverage index and their associations with willingness to receive the COVID-19 vaccine across racial/ethnic groups in the U.S
Source: Front Public Health. 2023 Oct 12;11:1192748. doi: 10.3389/fpubh.2023.1192748 (PMC10602638; doi:10.3389/fpubh.2023.1192748)
Supplement: Supplementary file 3 [file Table_3.pdf]

Table S3. Adjusted multigroup model comparisons of fully constrained versus freely estimated models

Model Parameter	Fully constrained model: 51 free parameters		Freely estimated model: 65 free parameters	p-value
**Overall county-level vaccination barriers**				
Log-likelihood value	- 17634.96		- 17619.71	
Scaling correction factor	1.50		1.48	
Scaled χ^2 difference		21.36		0.09
**Sociodemographic barriers**				
Log-likelihood value	-17629.48		- 17623.80	
Scaling correction factor	1.50		1.51	
Scaled χ^2 difference		7.38		0.92
**Limited healthcare system resources**				
Log-likelihood value	-17633.16		- 17627.48	
Scaling correction factor	1.50		1.49	
Scaled χ^2 difference		7.71		0.90
**Healthcare accessibility resources**				
Log-likelihood value	-17634.31		- 17621.34	
Scaling correction factor	1.49		1.46	
Scaled χ^2 difference		19.34		0.15
**Irregular care-seeking behavior**				
Log-likelihood value	-17630.77		- 17618.94	
Scaling correction factor	1.50		1.50	
Scaled χ^2 difference		15.55		0.34
**History of Low Vaccination**				
Log-likelihood value	-17634.47		- 17613.86	
Scaling correction factor	1.51		1.48	
Scaled χ^2 difference		28.86		0.01

SBSDiff package uses the loglikelihood values from nested models estimated using the maximum likelihood with robust standard errors (MLR) estimator, the number of free parameters, and the scaling correction factor for MLR to provide the Satorra-Bentler scaled change in model chi-square (χ2) and the p-value associated the with the χ2 difference.

Interpretation: The scaled χ2 difference represents the difference in model fit between the fully constrained model (i.e., model in which associations between the county-level vaccination barriers and willingness to receive the COVID-19 vaccine were held equal across racial/ethnic groups) compared to the freely estimated models (i.e., model in which associations between the county-level vaccination barriers and willingness to receive the COVID-19 vaccine allowed to vary freely across racial ethnic groups). Significant χ2 differences indicates significant interactions between the county-level vaccination barrier and race/ethnicity in these models.
